# Aging and sinus node dysfunction: mechanisms and future directions

**DOI:** 10.1042/CS20231025

**Published:** 2025-06-11

**Authors:** Thassio Mesquita, Rodrigo Miguel-dos-Santos, Eugenio Cingolani

**Affiliations:** Cedars-Sinai Medical Center, Smidt Heart Institute, 8700 Beverly Boulevard, Davis Building, Los Angeles, CA 90048, U.S.A.

**Keywords:** aging, cardiac arrhythmia, cardiac conduction system, sinoatrial node, sinus node dysfunction

## Abstract

Aging is a natural biological process influenced by endogenous and exogenous factors such as genetics, environment, and individual lifestyle. The aging-dependent decline in resting and maximum heart rate is a conserved feature across multiple species, including humans. Such changes in heart rhythm control underscore fundamental alterations in the primary cardiac pacemaker, the sinoatrial node (SAN). Older individuals often present symptoms of SAN dysfunction (SND), including sinus bradycardia, sinus arrest, and bradycardia-tachycardia syndrome. These can lead to a broad range of symptoms from palpitations, dizziness to recurrent syncope. The sharp rise in the incidence of SND among individuals over 65 years old, coupled with projected longevity over the next decades, highlights the urgent need for a deeper mechanistic understanding of aging-related SND to develop novel and effective therapeutic alternatives. In this review, we will revisit current knowledge on the ionic and structural remodeling underlying age-related decline in SAN function, and a particular emphasis will be made on new directions for future research.

## Introduction

The sinoatrial node (SAN) is the primary pacemaker of the heart, responsible for the initiation of rhythmic electrical impulses that lead to synchronized cardiac contractions. Located in the right atrium at the junction of the crista terminalis and the superior vena cava [1], the SAN consists of a small cluster of highly specialized pacemaker cells that generate spontaneous action potentials. The automaticity of these pacemaker cells is a key feature that distinguishes them from other cardiac myocytes. This unique electrogenesis allows the human heart to beat over two billion times throughout an average lifetime. However, when the SAN fails to self-generate electrical impulses, it compromises the systemic circulation leading to dizziness and syncope. Early-stage SAN dysfunction (SND) may be latent and asymptomatic, while more severe cases demand the implantation of an electronic pacemaker.

SND can arise from a variety of conditions, broadly categorized into genetic and acquired causes. Genetic factors include inherited conditions such as sick sinus syndrome, where specific gene mutations result in structural and/or functional abnormalities in the SAN tissue [2-5]. Mutations in several genes, including those encoding ion channels such as hyperpolarization-activated cyclic nucleotide-gated 2 and 4 (*Hcn2* and *Hcn4*) and sodium voltage-gated channel alpha subunit 5 (*Scn5a*), as well as cytoskeletal proteins and transcription factors involved in heart development, have been linked to SND [3,6,7]. For a comprehensive review of the genetic mechanisms underlying SND, we recommend readers see references [2,3,6]. However, most SND cases are due to non-genetic factors including, aging [8-10], heart failure (HF with reduced and preserved ejection fraction) [11-16], amyloidosis [17], systemic sclerosis [18,19], rheumatic fever [20], Chagas disease [21,22], and secondary to chemotherapies including immune checkpoint inhibitors [23-25].

**Figure 1:**
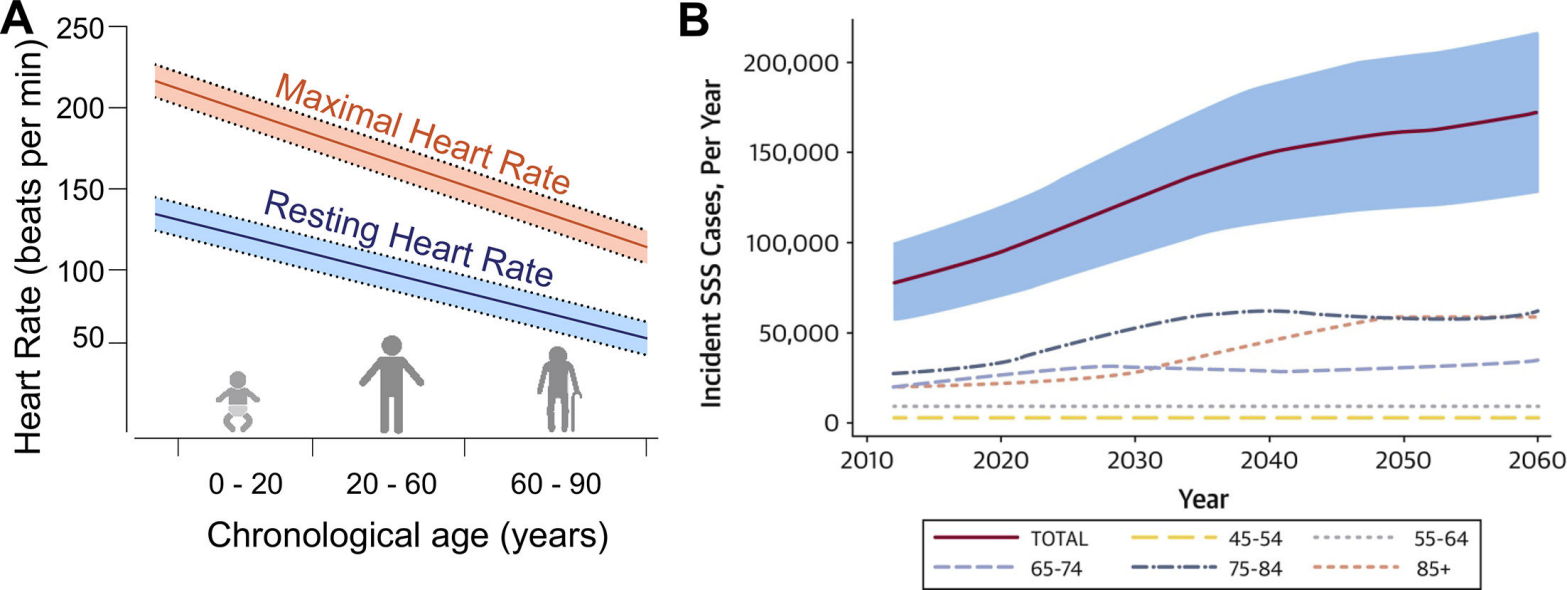
Age-dependent SND. (**A**) Decline in intrinsic and maximum heart rates with age. (**B**) Estimative of overall number of sick sinus syndrome (SSS) cases per year and stratified by age (United States, 2012–2060; reproduced with permission from [7]). Part of panel A was created with BioRender.com. SAN, sinoatrial node; SND, SAN dysfunction.

Aging-related SND is a common cardiac condition characterized by a progressive decline of SAN function (Figure 1A), impairing its ability to accurately regulate heart rhythm. SND affects approximately 1 in every 600 individuals over the age of 65 [7,26] (Figure 1B). According to the World Population Prospects 2024 from the United Nations, the current global population of people aged ≥80 years is 137 million, a number projected to triple by 2050 [27]. As human lifespans continue to increase, SND has become an increasingly relevant clinical problem, especially since current treatment options are limited to the costly and not risk-free implantation of electronic pacemakers. Therefore, a better understanding of the cellular and molecular mechanisms involved in aging-related SND could potentially generate new alternative therapeutic approaches for aging and other diseases that manifest with SND. While previous reviews have discussed important aspects of aging-related SND [28,29], the next section will provide a brief overview of the major ionic and structural remodeling that occur in SAN during aging.

## Physiology and pathophysiology of aging-related SND

In the mammalian heart, three major structures are endowed with automaticity and are capable of driving the heartbeat: the SAN, which serves as the primary pacemaker, the atrioventricular node (AVN), and the Purkinje fibers network. In 1882, Walter Gaskell demonstrated in the tortoise heart that cardiac electrical impulses are generated in the sinus auricle and then conducted to the atrium and ventricles [30,31]. A more detailed anatomical localization of the SAN pacemaker was later identified by Arthur Keith and Martin Flack in 1907 [32].

**Figure 2:**
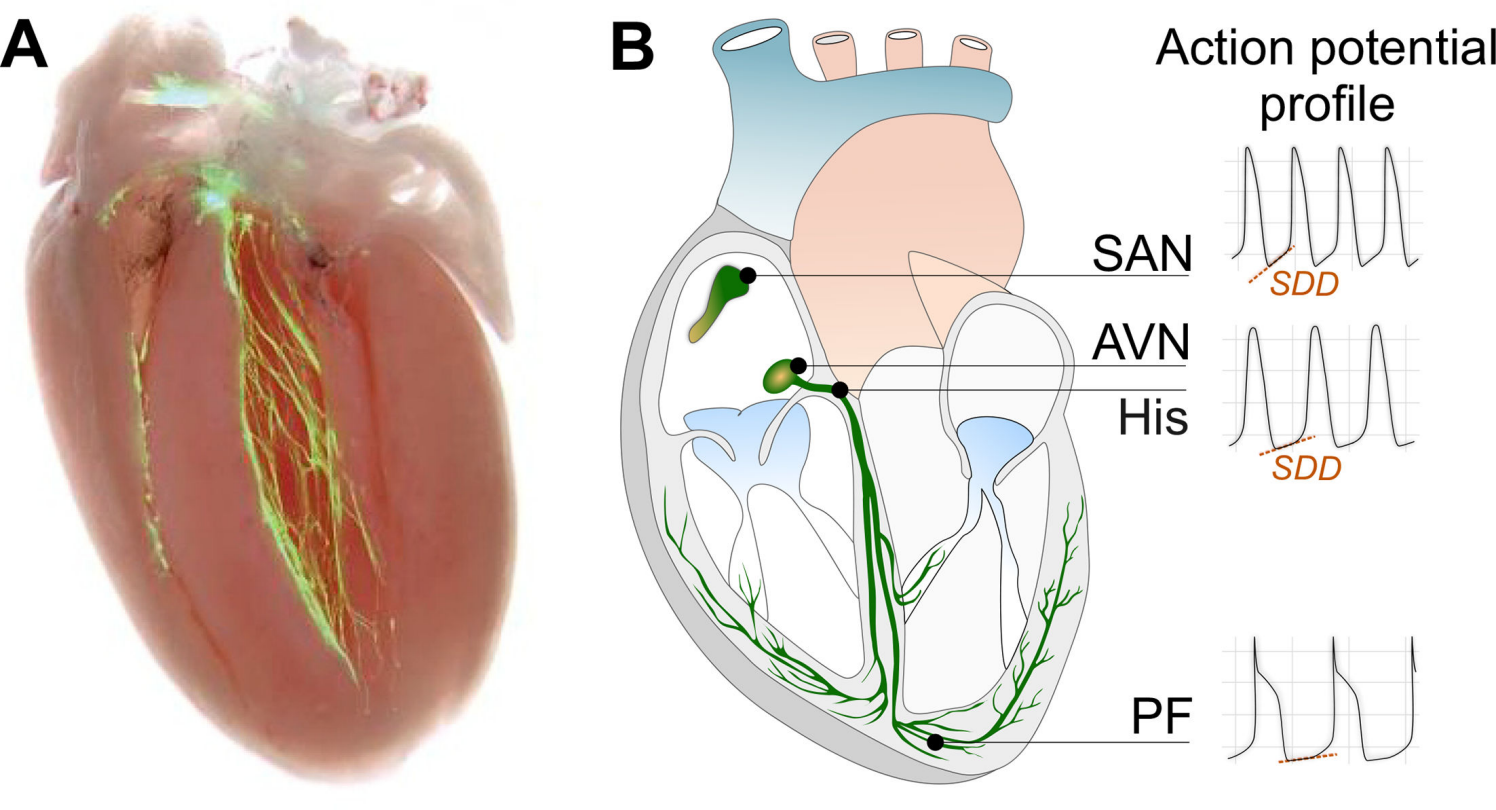
Cardiac conduction system. (**A**) Cardiac conduction system visualized using contactin2-eGFP mice (reproduced with permission from [33]). (**B**) Schematic representative anatomy of the cardiac conduction system. The cardiac electric impulse originates in the sinoatrial node (SAN) and travels across the atrioventricular node (AVN), the His bundle, left and right bundle branches, and Purkinje fibers (PFs) (reproduced with permission from [34]).

SAN pacemaker cells are specialized cell types that spontaneously oscillate their membrane potential, generating rhythmic electrical impulses that travel through the cardiac conduction system (CCS) to stimulate the entire heart (Figure 2). These electrical impulses quickly spread to the right and left atria, slowing down when reaching the AVN. This delay, along with the decremental property of the AVN, allows the atria to contract, completing the filling of ventricles with blood in preparation for their contraction at different heart rates (HRs). Subsequently, electrical impulses travel through the left and right bundle branches, which rapidly propagate throughout the ventricles via the Purkinje fibers to produce a synchronized biventricular contraction. Although pacemaker cells are distributed throughout the CCS, SAN cells exhibit a higher rate of spontaneous depolarizations compared with those in the AVN, His bundle, bundle branches, and Purkinje fibers, establishing the SAN as the primary pacemaker of the heart (Figure 2B).

**Figure 3:**
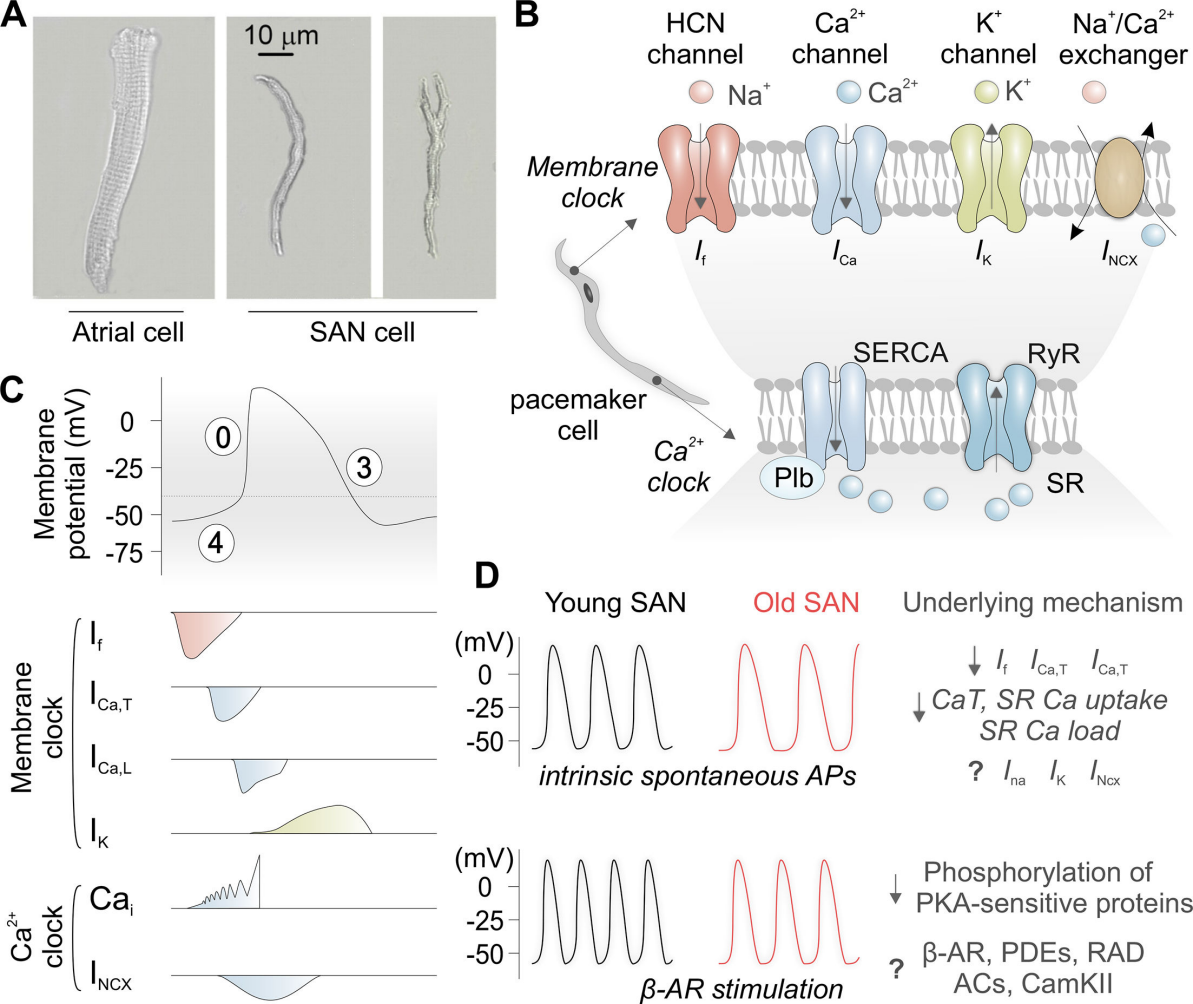
Depressed automaticity of aged SAN pacemaker cells. (**A**) Diverse morphology of pacemaker cells within the canine SAN tissue (center: spindle cell; right, spider cell; Reproduced with permission from [39]). (**B**) The coupled-clock system generates spontaneous diastolic depolarizations by functional interplay between ion channels at the plasma membrane (membrane clock) and local diastolic Ca^2+^ release (calcium clock). (**C**) SAN pacemaker cell action potential and ionic fluxes responsible for automaticity (B and C, adapted and reproduced with permission from [34]). (**D**) Reduced intrinsic spontaneous action potential (AP) firing rate and impaired β-adrenergic receptor (β-AR) response in aged SAN cells compared to young. AC, adenylyl cyclases; CamKII, Ca^2+^/calmodulin-dependent protein kinase II; PDE, phosphodiesterases; PKA, protein kinase A; SR, sarcoplasmic reticulum.

The automaticity of SAN pacemaker cells relies on the synchronized activity of two oscillators [34-37], referred to as the ‘membrane clock’ and the ‘calcium (Ca^2+^) clock’. Although SAN pacemaker cells are morphologically diverse [38,39] (Figure 3A), they are capable of generating rhythmic depolarizations with a unique action potential profile. Briefly, the membrane clock operates within the plasma membrane and relies on the hyperpolarization-activated funny current (*I*_f_), which provides an inward current during diastolic depolarization through the Hcn1, Hcn2 and Hcn4 channels [37,40-44]. As the membrane potential depolarizes, activation of T-type calcium channels (mainly Ca_v_3.1 and Ca_v_3.2) and l-type calcium channels (primarily Ca_v_1.2 and Ca_v_1.3) takes place, contributing to inward calcium currents (*I*_Ca_) [37,45-48]. The influx of ions from *I*_f_ and *I*_Ca_ synchronizes the rhythmic Ca^2+^ release from the sarcoplasmic reticulum via ryanodine receptors (RyR) [49-51], facilitating diastolic depolarization through the electrogenic sodium–calcium exchanger (NCX) [52,53]. The repolarization of the membrane potential is achieved through rapid and slow delayed rectifier potassium currents (*I*_Kr_ and *I*_Ks_, respectively), with additional contributions from *I*_K,ACh_, *I*_K,ATP_, *I_K_*_,Ado_, and *I_K_*_,Ca_ [37,54-59]. Subsequently, Ca^2+^ reuptake into the sarcoplasmic reticulum by the sarcoplasmic reticulum (SR) Ca^2+^ ATPase (SERCA) takes place, which, in combination with repolarizing currents, reestablish the maximum diastolic membrane potential to facilitate the initiation of a new heartbeat (Figure 3B and C). For more details on the numerous ionic currents involved in cardiac pacemaking, we encourage readers to consult the following reviews [37,60]. Although not discussed in detail here, alterations in calsequestrin 2 [61], junctophilin 2 [62], desmosomal proteins (e.g., desmoplakin and desmin) [63,64], natriuretic peptides receptors (NPR-A, NPR-B, and NPR-C) [65-68], Hippo-Yap signaling [69], glucagon-like peptide-1 receptor [70], p21-activated kinase 1 [71], phosphodiesterases [72-76], cyclic guanosine monophosphate, and protein kinase D [77] have been linked with the control of SAN function in healthy and diseased hearts.

The gradual decline in HR with aging reflects a slowing of the intrinsic SAN activity (Figure 3D), resulting from both electrical remodeling of individual pacemaker cells and structural changes within the SAN tissue. Aging-dependent ionic remodeling of SAN is considered a key mechanism that explains the reduced automaticity of pacemaker cells. A decrease in the densities of *I*_f_, *I*_Na_, *I*_Ca,L_, and *I*_Ca,T_ occurs in an age-dependent manner [78-81], thereby contributing to lower action potential firing rates in aged SAN. Despite the clear role of *I*_NCX_ and *I*_K_ in pacemaker function, there is currently no functional evidence regarding their contributions in aged SAN cells. Changes in several Ca^2+^-handling proteins have been reported in aged SAN, suggesting an impairment of the Ca^2+^ clock. The expression levels of RyR2, SERCA2a, and NCX1 are decreased in aged SAN cells [82,83], which partially justifies the reduced Ca^2+^ transient amplitude, prolonged SR Ca^2+^ reuptake, and consequently, SR Ca^2+^ load [82]. Although the cellular components of membrane and Ca^2+^ clocks have been considered as spatially distinct [84], there is compelling evidence highlighting the requisite of a coupled-clock system to maintain the automaticity of SAN pacemaker cells [35]. Thus, further studies are still required to further dissect the precise mechanisms driving the ‘uncoupled’-clock system in aged SAN cells.

Chronotropic incompetence is defined as the inability of the heart to accelerate its rate to adequately match cardiac output to the increased body’s demand [85,86]. This condition is considered a major limiting factor in exercise capacity, which limits the quality of life and serves as an independent predictor of major adverse cardiovascular events and overall mortality [85,86]. Although the exact mechanisms of chronotropic incompetence have yet to be elucidated, among other factors [85,86], SND is considered an important contributor. During strenuous exertion, the HR can be increased by up to 300%; however, a blunted maximum HR in response to exercise and sympathetic stimulation is observed in older individuals [85-87]. This acute chronotropic adaptation is a mechanism modulated by the autonomic nervous system, which releases catecholamines by sympathetic neurons. These neurotransmitters activate the β-adrenergic receptor (βAR), a Gs protein-coupled receptor located at the membrane of SAN pacemaker cells, with subsequent augmented intracellular levels of cyclic adenosine monophosphate (cAMP) via adenylyl cyclase [88,89]. cAMP directly binds to HCN channels increasing *I*_f_ [90] and stimulates protein kinase A (PKA), which phosphorylates numerous proteins that increase the firing rate of SAN cells [82,91-95]. The impaired response of aged SAN to βAR stimulation is partly justified by the altered expression of PKA-sensitive proteins. So far, no age-dependent changes in βARs transcription have been found in SAN [83,96]. Most previous research in aged SAN has focused on dissecting the cellular responses of components downstream of βAR signaling, revealing that stimulating adenylyl cyclase or inhibiting phosphodiesterases increases firing rates in both young and aged SAN cells [78,79]. While the relative increase in firing rates in young and aged SAN was similar, the absolute number of action potentials generated by aged SAN cells was significantly lower [78,79] (Figure 3D). Interestingly, the replenishment of intracellular concentrations of cAMP restored the firing rate in aged SAN to young levels [79]. These findings suggest that the impaired fight-or-flight response in aged SAN cells is more closely associated with reduced intracellular cAMP production than changes in βAR expression.

## Aging-related structural remodeling of SAN

The SAN tissue is unique in its cellular and extracellular matrix composition (ECM) [97,98]. Pacemaker cells within the SAN are connected to each other via gap junctions, primarily connexin 45 (Cx45) and connexin 30.2 (Cx30.2), unlike the working myocardium where connexin 43 (Cx43) is more commonly expressed. Along with the particular ECM microstructure of SAN tissue discussed below, the pattern of gap junctions contributes to the functional connection between the SAN and atria myocardium through SAN conduction pathways (SACPs) [99-101]. Age-related slowing of SAN conduction has been observed [102,103], with studies reporting a progressive reduction in the expression of Cx43 and Cx30.2 without significant changes in Cx40 or Cx45 [83,102]. Given the naturally low levels of Cx43 in the SAN, the link between slowing SAN conduction and Cx43 expression requires further exploration.

The ECM of the SAN is notably distinct from other compartments of the heart, containing a high content of collagen, elastin, and fibroblasts [97,99-101,104,105]. This rich network of connective tissue not only supports the structural integrity of the SAN but also functions as an electrical insulator, regulating the velocity and directionality of electrical impulse propagation and ensuring that the SAN remains the dominant pacemaker of the heart [97,106]. Increased fibrosis within the SAN has been widely observed across various species and is strongly associated with reduced pacemaker function [8,107-110]. This architectural remodeling disrupts electrical conduction, with more pronounced effects seen in older individuals with SND [10,111]. However, while fibrosis is commonly linked to pacemaker abnormalities, its precise role in the age-related slowing of intrinsic HR remains uncertain. Interestingly, fibrotic remodeling is not always correlated with SND, as some aged hearts with significant fibrosis maintain normal sinus rhythm [107]. Therefore, the extent to which fibrosis contributes to the decline of SAN function with age has yet to be determined.

## Management of SND and aging-related SND

**Figure 4:**
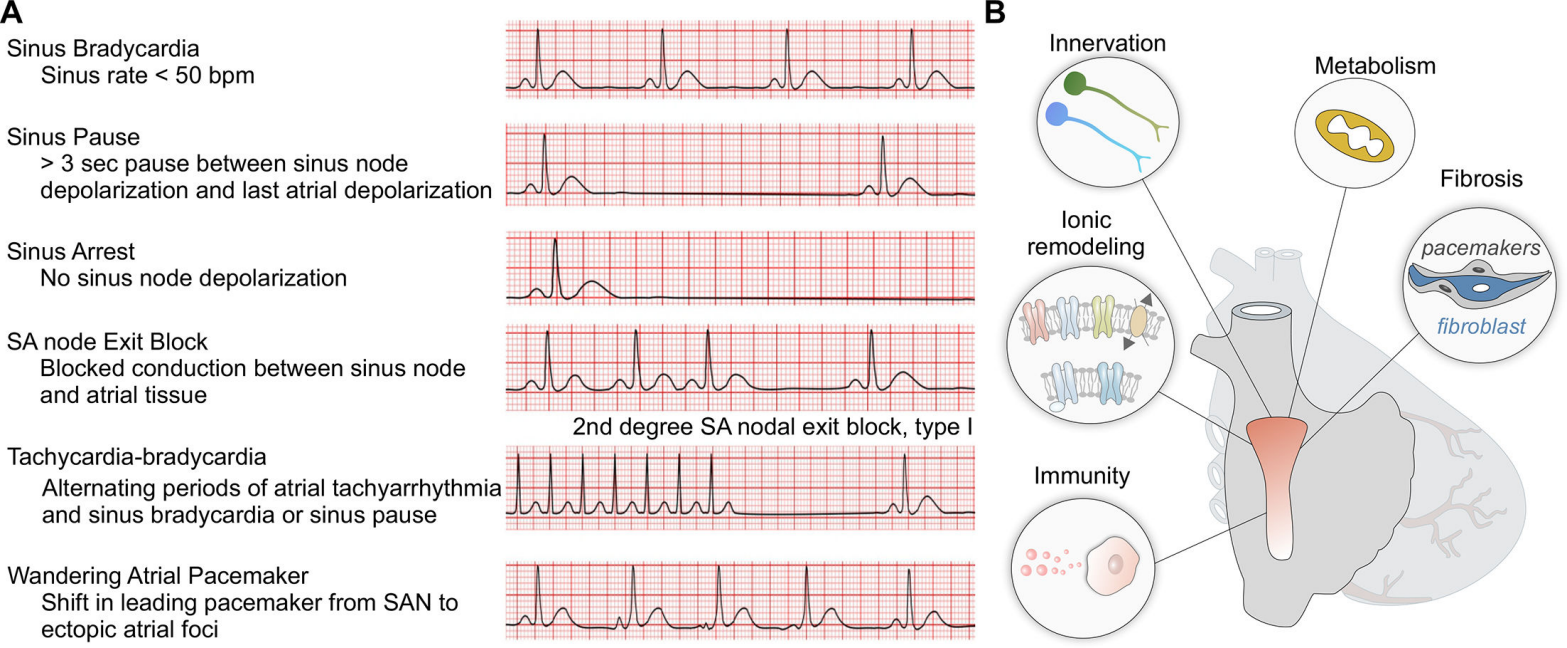
Postulated mechanisms of aging-related SND. (**A**) Classifications of SND (reproduced with permission from [2]). (**B**) Underlying mechanisms associated with aging-dependent SND. SAN, sinoatrial node; SND, SAN dysfunction.

The management of symptomatic patients with SND focuses on alleviating symptoms and preventing complications (Figure 4A), such as syncope, dizziness, or HF. Acute and reversible SND cases are commonly managed with medications or temporary transvenous or transesophageal pacing [2,112,113]. However, most cases of aging-related SND are chronic and irreversible [26,112]. Unfortunately, there are currently limited options for treating chronic SND, with the implantation of a permanent electronic pacemaker remaining the primary treatment option. The Dutch registry data from 96,900 patients demonstrated that 32.6% of individuals aged 80 or older necessitate pacemaker implantation, with SND the most common cause of indication (42.3%) [114]. In a larger cohort, the analysis of 178,000 pacemakers implanted in the United States between 1997 and 2004 indicated that 64% of all implantations were in patients aged 75 years or older [115]. Although benefits on the quality of life were observed in elderly patients treated with a permanent pacemaker, particularly using dual-chamber rate-modulated pacing [116], there are conflicting findings on survival benefits [26,117-121]. For a detailed discussion on electronic devices and biological pacemakers, we recommend reading the following review [122].

Multiple pharmacological agents have been shown to affect SAN physiology. Table 1 briefly summarizes the most clinically relevant drugs that affect the chronotropism of the heart and their mechanisms of action. Despite the therapeutic benefits, drug intoxication has been reported with consequent effects on the sinus rhythm, as previously discussed [113]. However, molecules without anticipated effects on SAN chronotropy such as glucagon-like peptide-1 receptor agonist were recently shown to increase the firing rate of pacemaker cells via phosphorylation of Ca^2+^ cycling proteins [70]. While the repurposing of drugs for SND remains largely unexplored, the development of synthetic molecules, biological and gene therapeutic approaches are areas that warrant further studies.the

**Table 1:** Summary of the most clinically relevant drugs that affect the heart rate.

Class of drug	Mechanism of action	HR effect
β-AR blocker (e.g., atenolol, propranolol)	β-AR antagonism	↓
Cholinergic blockers (e.g., atropine)	muscarinic receptor antagonism	↑
Methylxanthine (e.g., theophylline)	Phosphodiesterase inhibitor and adenosine receptors blocker	↑
Benzazepine (e.g., ivabradine)	HCN blocker	↓
Digitalis glycoside (e.g., digoxin)	Na^+^/K^+^ ATPase inhibitor	↓
Ca^2+^ channel blockers (e.g., verapamil)	Ca^2+^ channel blockers	↓
K^+^ channel blockers (e.g., amiodarone)	K^+^ channel blockers	↓

Additionally, bioinformatic pipelines that analyze transcriptional signatures alongside predicted drug effects—using databases of bioactive molecules with drug-like properties [98,123]—could accelerate drug repurposing for SND. While these predictions require experimental validations, advances in data mining are necessary to enhance their reliability. For instance, integrating multiple bioactive molecule databases may empower the discovery of new classes of drugs or analogs. Furthermore, incorporating disease-associated transcriptional signatures and the introduction of artificial intelligence methods such as machine learning in combination with proteomics and metabolomics studies may further increase the accuracy of predictions and expand the therapeutic options for SND.

Understanding the molecular mechanisms and pathways involved in the development of SND holds the potential to uncover novel therapeutic targets (Figure 4B), paving the way for more effective and patient-specific therapies. Much of our current knowledge about the molecular mechanisms governing the SAN function has been derived from animal models, mainly due to practical reasons. Despite numerous structural and functional commonalities across multiple species, differences exist and should be interpreted with caution.

## Emerging investigation areas

To develop more effective and alternative therapeutic strategies for SND, future research must prioritize a deeper exploration of the cellular and molecular mechanisms that underlie the condition. In this section, we provide our perspective on key topics that deserve further investigation in the context of aging-related SND (Figure 4B). Given the numerous underexplored areas, we emphasize that the mechanisms discussed below are just a few among many that require further studies.

### Mechanosensitivity of SAN pacemaker cells

As previously mentioned, voltage-gated ion channels are crucial for SAN automaticity. In addition, pacemaker cells are equipped with a variety of mechanosensitive ion channels that influence their automaticity [124]. A landmark study by Cooper et al. [125] demonstrated that moderate stretching (5–10% of resting cell length) of isolated SAN cells led to an increased beating rate, suggesting that pacemaker cells functionally encode mechanosensitive mechanisms that modulate their chronotropic response.

The positive chronotropic response induced by sustained stretch seems to be linked to the activation of quiescent nodal cells [126]. Interestingly, prolonged stretching (up to 5 min) of SAN elicits a biphasic response, characterized by an immediate acceleration of beating rate followed by a gradual return to pre-stretch levels [127]. This reversible stretch-induced behavior may arise from the activation of slow mechanosensitive channels and mechanochemical signaling pathways [124,128-130]. These ion channels can be classified into two categories: fast (within seconds), directly mechanoactivated (Piezo1-2, TREK-1, TRAAK, and BK) and slow (within minutes), indirectly mechanoresponsive (TRP channels, LRRC8, and ClC). In murine SAN, transcripts for Piezo1, LRRC8a, ANO1, TASK-1, and TRPM7 were found to be more abundant than HCN4 [131]. Although the precise mechanisms by which mechanical forces alter the chronotropic response are not fully understood, it is likely that the activation of mechanochemical pathways, which generate intracellular second messengers like reactive oxygen species, cAMP, and inositol trisphosphate, modifies Ca^2+^ handling and ion channel activity within the SAN.

Increased and sustained intra-atrial pressures are known contributors to the development of ectopic foci and atrial arrhythmogenesis [132-134]. However, there is limited information regarding the causal role of mechanosensitive pathways in SND. Transcripts for TREK1 and TASK1 were found to be upregulated in aged compared to young [83]. A recent deep RNA sequencing performed on aged and young SAN tissues provides a valuable resource for further exploration into whether other mechanoactivated channels are also altered by aging [135]. While the transcriptional landscape offers insights, the mechanisms driving these transcriptional changes—and, more importantly, the functional implications of mechanoactivated channels in aging-related SND—remain largely unexplored. Given that mechanical forces can be influenced by fibrotic remodeling of aged SAN tissue, elucidating the precise mechanisms involved poses significant experimental challenges. In summary, the interplay between mechanoelectrical and mechanochemical signals within SAN tissue introduces a layer of complexity to the regulation of pacemaker automaticity, forming a novel concept of a ‘mechano clock’ that may affect the dual voltage-Ca^2+^ clocks. Therefore, a deeper understanding of the regulation, localization, and function of these pathways is essential for considering them as potential therapeutic targets for SND.

### Heterogeneity of SAN pacemaker cells

The SAN is a heterogeneous tissue composed of a variety of cell types [98,136]. Sub-specialized pacemaker cells have been classified into three morphologically distinct cells: elongated spindle, spindle, and spider cells [38]. Pacemaker cells are densely packed within the core region of the SAN, initiating the spontaneous depolarizations that set the rhythm of the heart. Characteristically smaller and spindle-shaped, these cells contain fewer myofibrils than neighboring atrial myocytes. Interestingly, a gradual transition in action potential properties from the central SAN area toward its periphery suggests the presence of transitional cells [137]. While not fully characterized, these transitional cells display intermediate characteristics between pacemaker cells and atrial myocytes. Unlike SAN cells from the core region, transitional cells may be interconnected by gap junctions [100]. Although a deep molecular and electrical understanding of each morphologically distinct pacemaker cells warrants investigation, the extent to which they individually contribute to SND in aging has yet to be determined.

A coordinated depolarization of a cluster of SAN pacemaker cells generates a relatively weak current flow, acting as a ‘source’ that diffuses into the adjacent atrial myocardium, which serves as the ‘sink’. This capacity of SAN currents to drive the overwhelming hyperpolarized potentials of well-coupled atrial myocardium highlights the intricate tissue architecture that is composed of specialized branching myofibers from SACPs [100,101,138]. Histological analyses have shown that the volume of SAN cells declines with age [139,140]. Additionally, cellular hypertrophy and increased membrane capacitance have been observed in aged SAN [78,139,140]. Thus, the numerical reduction of SAN pacemaker cells as a result of aging may limit the cluster of electrically coupled cells needed to effectively excite the surrounding atria myocardium, leading to a source-sink mismatch. Although aging is linked with the activation of multiple signaling pathways that promote cell death [141], determining the mechanism mediating the aging-related pacemaker cell loss may open avenues for new therapeutic targets. Moreover, although structural remodeling also occurs in aged SAN, further investigation is needed to determine whether the formation of discontinuous myofiber tracts in specific regions of SACPs is causally related to conduction blocks in aged SAN tissue.

### Non-canonical roles of macrophages in SAN function

Many arrhythmic disorders are associated with an inflammatory component. We and others have demonstrated that excessive production of inflammatory cytokines and chemokines contributes to the development of arrhythmogenic substrates [142-147]. Recent discoveries suggest that immune cells can also influence the cardiac rhythm by non-canonical electrotonic coupling with cardiomyocytes [142,148]. Tissue-resident macrophages are the most abundant immune cell type in rodent and human hearts (≈5% total cells), playing a wide range of physiological and pathological roles [149-151]. Single-cell RNA sequencing of mice hearts has identified three transcriptionally distinct macrophage subsets: TLF^+^ macrophages (expressing *Timd4*, *Lyve1*, and *Folr2*, but lacking *Ccr2* and low MHC-II expression), MHC-II^hi^ macrophages (lacking *Timd4*, *Lyve1*, *Folr2*, and *Ccr2*), and CCR2^+^ macrophages (lacking *Timd4*, *Lyve1*, and *Folr2*, but high MHC-II expression) [149-151]. This diversity clarifies the biological distinctions between self-renewing resident macrophages (CCR2^−^) and monocyte-dependent macrophages (CCR2^+^), shedding light on their specific roles during cardiac injury [149,152,153].

The role of macrophages in the CCS remains largely unexplored. Macrophages were previously shown to directly couple with pacemaker cells in the AVN facilitating the electrical conduction via a Cx43-dependent mechanism [148]. However, to our knowledge, no previous research has specifically addressed the role of resident macrophages in regulating SAN function. In this context, a recent study combined single-nuclei RNA sequencing and spatial transcriptomics to analyze human SAN tissue from individuals without a history of cardiac disease or arrhythmia [98]. Three distinct populations of tissue-resident macrophages were identified, including *Lyve1* ^+^ *Igf1^+^*, *Lyve1^+^Timd4^+^*, and *Lyve1^+^* cycling. However, their functional role has yet to be established. Intriguingly, Cx43 or other connexins were minimally expressed in macrophages present in SAN and AVN [98]. Therefore, the mechanisms by which the depletion of resident macrophages, using Cd11b-expressing diphtheria toxin receptor mice, leads to third-degree AV block remain to be further investigated.

In isolated cardiac resident macrophages, four patterns of outward and two patterns of inward-rectifier potassium currents have been described [154]. K_v_1.3, K_v_1.5, and Kir2.1 were abundantly expressed in macrophages [154], likely contributing to the observed ion currents and potentially influencing cardiac excitability. Given the presence of distinct macrophage subsets in the heart, further research is needed to determine if all these identified subsets similarly affect cardiac electrophysiology. In a mouse model of HF with SND, significant remodeling of the inflammatory proteome was observed [155]. A notable increase in CCR2-expressing macrophages was observed in the SAN of failing hearts, while systemic inhibition of the pro-inflammatory galectin-3 improved the SND [155]. However, this area of research remains largely unexplored in the context of aging-related SND. This knowledge gap highlights the need for future studies to investigate whether resident and/or recruited macrophages contribute to aging-related SND, and to uncover the mechanisms through which they may influence pacemaker function.

### Regulation of SAN gene expression

The understanding of the early development of the SAN may reveal molecular insights into a potential loss of gene expression programming in aged pacemaker cells. The first spontaneous Ca^2+^ oscillations occur in the mouse embryo at around E7.75 [156,157], initiating the uninterrupted automaticity that sustains heart function throughout life. This critical event is the result of a finely orchestrated regulation of transcription factors that guide the differentiation and maturation of pacemaker cells [158-160]. During early cardiogenesis, T-box transcription factors Tbx5, Tbx18, and insulin gene enhancer protein ISL1 give rise to the sinus venosus myocardium. Tbx5 activates short stature homeobox protein 2 (Shox2) and Tbx3, which along with Isl1, regulate the SAN pacemaker gene program [158-160]. Shox2 also inhibits the expression of the homeobox protein NKX2.5, which along with Tbx3 and pituitary homeobox 2 protein (Pitx2) determine the formation of the SAN region and prevents its ‘atrialization’ [6,158].

Transcriptomic analysis of aged SAN tissue revealed an up-regulation of Tbx3 and Tbx5 compared with young counterparts, consistent with increased expression of pacemaker-related genes HCN1 and HCN4 [135]. Despite this, a reduction in *I*_f_ has been consistently reported in aged SAN cells, pointing to complex gene expression regulatory mechanisms in aged pacemaker cells that require further investigation. One limitation of many current studies using next-generation RNA sequencing is the lack of transcriptional changes at single-cell resolution. Pacemaker cells are considered to be large in size for conventional droplet-based platforms, with most of the single-cell RNA sequencing data obtained from SAN cells during embryonic or neonatal stages [161,162]. However, single-nuclei RNA sequencing and newer technologies such as plate-based platforms and spatial transcriptomics can circumvent cell size limitations [98,136]. Given the heterogeneity of pacemaker cells in the SAN [38], embracing the patch-sequencing technology may connect distinct morphology and electrophysiology of pacemaker cells with unique transcriptomic signatures [163].

In recent decades, research on non-coding RNA molecules, particularly microRNAs (miRNAs), has highlighted their critical role as regulators of mRNA and protein expression [164]. Approximately 30% of protein-coding genes are influenced by miRNAs [165], with their expression and function undergoing dynamic changes during development and various pathological conditions [166,167]. miRNAs also regulate genes essential for cardiac pacemaking. Profiling of miRNAs in human SAN tissue has revealed a distinct expression pattern compared to the right atrium, with many SAN-enriched miRNAs predicted to target key pacemaking genes including, *Hcn4*, *Cacna1c*, *Cacna1d*, *Kcnj3, Kcnj5, and Ryr2* [168,169]. A summary of the miRNA interaction with ion channels in the SAN is provided in Table 2. While this list focuses on findings from SAN tissue, a broader list of ion channels modulated by miRNAs in atrial and ventricular cells can be found elsewhere [173,174].

**Table 2:** miRNAs involved in the regulation of genes related to pacemaking function.

	microRNA in the SAN	Genes	Ref.
*I*_Na_	miR 153–3 p, miR-486–3p, miR-92a-3p, miR-652–5p, miR-3200–3p, let-7g-3p, mir-1180–3p	*Scn8a, Scn5a*	[168,169]
*I*_f_	miR-1, mir-30c-5p, miR-133–3p, miR-187–3p, miR-211–5p, miR-370–3p, miR-423–5p, miR-486–3p, miR-652–5p, miR-3200–3p	*Hcn1, Hcn2, Hcn4*	[168-171]
*I*_K_	mir-1247–5p, mir-30c-5p, mir-486–3p, mir-423–5p, mir-1260-a, mir-744–5p, mir-193a-3p, mir-574–5p, mir-133a-3p, mir-215	*Kcnj3, Kcnj5, Kcnj11, Kcne1, Kcnq1, Kcna4*	[168,169]
*I*_Ca,L_	miR 30 c‐5 p, miR-1976, mir-198, mir-153–3p, mir-204–5p, mir-371–3p, mir-938	*Cacna1c, Cacna1d*	[168,169,172]
*Ryr*	mir-198, mir-153–3p	*Ryr2*	[168]
*I*_NCX_	miR-1–3p	*Slc8a1*	[169]

There is compelling evidence that miRNA-based therapeutics may offer promising avenues for the treatment of SND. For instance, the down-regulation of miR-423–5p has been shown to reverse exercise training-induced remodeling of the Hcn4 channel, alleviating sinus bradycardia [175]. Additionally, local delivery of anti-miR-370–3p successfully restored *I*_*f*_ in a mouse model of HF [170]. Furthermore, our recent research revealed that chemically modified human Tbx18 mRNA is suppressed by endogenous miRNAs. By employing counterstrategies to inhibit these suppressive miRNAs, we were able to achieve robust expression of Tbx18 protein, resulting in increased automaticity in a rat model of AV block [176].

While miRNAs have been implicated in regulating age-related processes across various mammalian tissues, their specific role in aging-related SND has yet to be investigated. Given that a single miRNA can influence hundreds of target genes involved in diverse signaling pathways, miRNA-based therapies could potentially lead to significant therapeutic benefits [166,167,177]. However, the possibility of off-target effects and adverse effects must be carefully considered. Thus, further research is needed to assess the viability and safety of miRNA-based therapies for cardiac rhythm disorders.

### Metabolic control of SAN pacemaker cells

Pacemaker cells from SAN tissue maintain a high basal cAMP-PKA signaling driven by Ca^2+^-dependent activation of adenylyl cyclase [51]. Despite the relatively low density of contractile myofilaments in SAN myocytes compared to ventricular myocytes [97], SAN cells consume more oxygen than ventricular myocytes paced at 3 Hz [178]. However, such elevated demand comes with a high energetic cost, requiring an efficient energy supply and consumption. The SAN is indeed equipped with a dense mitochondrial network with a high basal respiratory rate [178,179]. It was previously shown that decreased production of adenosine triphosphate (ATP) in SAN cells suppressed the automaticity by interfering with basal Ca^2+^-cAMP/PKA [178] or Ca^2+^-calmodulin-dependent protein kinase II CaMKII [180] signaling. In contrast, when the HR increases, such as during β-AR stimulation, ATP production must rise to meet the higher energy demand needed to sustain the elevated firing rate. In this context, the mitochondrial calcium uniporter revealed important for the acceleration of HR by facilitating Ca^2+^ entry into the mitochondrial matrix to enhance energy production via oxidative phosphorylation [181]. A significant remodeling of mitochondria with disruption of mitochondria–SR microdomains was recently described in the SAN of HF mice [179]. Down-regulation of mitofusin-2 was mechanistically linked with impairing mitochondrial function, energy production, and, consequently, SND [179]. AMP-activated protein kinase (AMPK) is a key cellular fuel sensor, detecting energy imbalances during metabolic stress [182], being the AMPK γ2 subunit the most abundant in the SAN [182]. Mice with gain-of-function mutation on R302Q γ2 exhibit sinus bradycardia due to reduced *I*_*f*_ and local SR Ca2+release in SAN cells [182]. Together, these findings reveal that energy homeostasis regulation in pacemaker cells is critical for preserving SAN automaticity.

The primary pacemaking site within the SAN region shifts in response to physiological stimuli, including activation by the autonomic nervous system [12,84]. Two spatially distinct pacemaker sites coexist within the SAN, identified in the superior and inferior anatomic regions of SAN tissue and preferentially regulating fast and slow HRs, respectively [84]. A recent investigation added an important layer of complexity by demonstrating the influence of anatomical variations of the local vasculature on SAN physiology [183]. High-resolution 3D reconstructions from intact SAN revealed that the superior site is highly vascularized and Hcn4-expressing pacemaker cells have more near contact with blood vessels compared with the inferior site [183]. These findings suggest that high vascular density in the superior SAN region may locally regulate nutrient supply to meet the SAN’s electrical demands. However, further studies are needed to determine whether microvascular rarefaction of SAN tissue occurs with aging. Additionally, the potential mechanistic link between aging-related SND and energy mismatch or metabolic inflexibility remains to be elucidated.

## Conclusions

Aging is a natural and unavoidable process that is often associated with SND. Despite significant progress made in understanding aging-related SND, electronic pacemaker implantation remains the only treatment option for these patients, regardless of the underlying mechanism. Extensive research has uncovered key age-related structural and functional changes in the SAN, which correlate with the age-dependent slowing of HR and SAN cells depolarizations. However, correlation does not imply causation, and further mechanistic studies are needed to clarify these connections. While we have highlighted some important gaps in knowledge, it is important to note that much of this research was conducted using preclinical animal models, such as rodents, pigs, and dogs due to practical and logistic reasons. While these models are valuable for mechanistic studies, they do not fully capture the complexity of human physiology, requiring cautious interpretation and conclusions. Additionally, there is a surprising lack of research on potential sex-dependent changes in the aging SAN, an area that warrants further investigation. Thus, expanding our understanding of these key identified areas, and others beyond the scope of this review, is crucial for ‘decoding the clock’ of aging hearts, reducing the reliance on electronic pacemakers, and developing innovative and disease-specific therapies for sinus node dysfunction in the elderly and related diseases.

## Abbreviations

AMPK: AMP-activated protein kinase
AVN: atrioventricular node
CCS: cardiac conduction system
CaMKII: Ca2+-calmodulin-dependent protein kinase II
Cav: voltage-gated calcium channel
ECM: extracellular matrix
HCN: hyperpolarization-activated cyclic nucleotide-gated channel
HF: heart failure
HRs: heart rates
Kv: Voltage-gated potassium channels
NCX: sodium-calcium exchanger
PKA: protein kinase A
SAN: sinoatrial node
SERCA: sarcoplasmic reticulum calcium ATPase
SND: SAN dysfunction
SR: sarcoplasmic reticulum
miRNA: microRNA
βAR: beta-adrenergic receptor
